# Construction and application of color fundus image segmentation algorithm based on Multi-Scale local combined global enhancement

**DOI:** 10.12669/pjms.37.6-WIT.4848

**Published:** 2021

**Authors:** Yanjie Hao, Hongbo Xie, Rong Qiu

**Affiliations:** 1 Yanjie Hao, Associate Chief Physician, Department of Ophthalmology, Jiaozhou Central Hospital of Qingdao, Qingdao, 266300, Shandong, China; 2 Hongbo Xie, Attending Physician, Department of Ophthalmology, Qingdao Women’s and Children’s Hospital, Qingdao, 266000, Shandong, China; 3 Rong Qiu, Associate Chief Physician, Department of Ophthalmology, Zhucheng People’s Hospital, Zhucheng, Shandong, China

**Keywords:** Color fundus image segmentation algorithm, Multi-scale line detector, Segmentation accuracy, Blood vessel

## Abstract

**Objective:**

Aiming at the problem of low accuracy in extracting small blood vessels from existing retinal blood vessel images, a retinal blood vessel segmentation method based on a combination of a multi-scale linear detector and local and global enhancement is proposed.

**Methods:**

The multi-scale line detector is studied, and it is divided into two parts: small scale and large scale. The small scale is used to detect the locally enhanced image and the large scale is used to detect the globally enhanced image. Fusion the response functions at different scales to get the final retinal vascular structure.

**Results:**

Experiments on two databases STARE and DRIVE, show that the average vascular accuracy rates obtained by the algorithm reach 96.62% and 96.45%, and the average true positive rates reach 75.52% and 83.07%, respectively.

**Conclusion:**

The segmentation accuracy is high, and better blood vessel segmentation results can be obtained.

## INTRODUCTION

Retinal blood vessels are the only part of the human body that can be directly observed non-invasively. By detecting changes in the structure of blood vessel width, angle, and branches, it can help diagnose diseases such as diabetes, glaucoma, and hypertension.^1^-^3^ The retinal vascular network is a tree-like structure with many branches, and the small blood vessels in the branches have a small contrast with the background, and the contour boundaries are blurred, which makes the automatic segmentation of small blood vessels more difficult.^4^-^6^

Therefore, this paper proposes a retinal vessel segmentation method based on a multi-scale linear detector combining local enhancement and global enhancement. First, the multi-scale linear detector is re-divided into two parts: small scale and large scale. The local and global enhanced images are then subjected to linear detection at small scale and large scale, respectively. Finally, detection at different scales is performed. Its results Fusion processing to get the final retinal blood vessel segmentation map.

## METHODS

Analysis of the original color retinal image shows that the contrast between the green channel image and the background is the largest, so the image after the green channel inversion is used for blood vessel extraction.

The linear detector defines a 15 × 15 window, using this window as a mask, calculates the average gray value 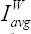 corresponding to any pixel point P in the retinal image, and then calculates the pixel point P as the center along the length L (L = 15), and find the average value 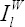 of the pixels passed by the line segment. With 15° as the step size, calculate 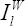 in 12 different directions. When the direction of the detection line is consistent with the direction of the blood vessel, 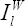 has a maximum value and is represented by 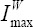. The response function of the blood vessel is thus obtained.


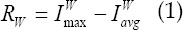


The maximum gray value of the pixel at the center of the detection line is not required when 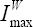 is maximum, which is why the basic linear detector can handle the central reflection of blood vessels. Due to the single scale and fixed detection line size, this method is prone to the following two problems:

1) Fusion two adjacent blood vessels into a single blood vessel.
2) Dilation occurs at the intersection of two blood vessels. Nguyen UTV Solved the above problem by changing L from 15 to 1, 3, 5, 7, 9, 11, 13, 15.

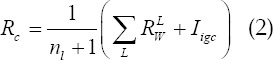


Where *n_L_* is the number of all scales, and *I_igc_* is the pixel value of the corresponding pixel of the original green channel image. Then use equation (2) to fuse the response functions at different scales to get the result. This method solves the dilation phenomenon that occurs when a blood vessel merges into a single blood vessel and intersects the blood vessels. However, this method applies all scales to the same image and does not preprocess the image differently according to different scales, resulting in loss of part of the image information and reduced the accuracy of blood vessel segmentation.

### Algorithm Description

Because small-scale highlights detailed information and large-scale highlights global information, different pre-processing of images for different scales can better extract blood vessel information. Therefore, in view of the problems existing in the above methods, this paper proposes the following methods.

### The steps are as follows:

**Fig.1:**
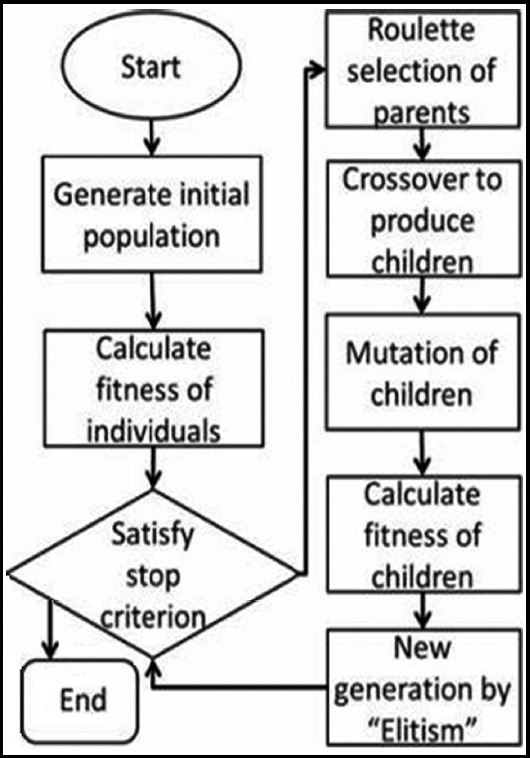
Algorithm flowchart.

1) re-dividing the multi-scale linear detector; 2) small-scale detection after local image enhancement and large-scale detection after global image enhancement; 3) image fusion processing stage at different scales. The specific algorithm flow is shown in Fig.1.

The study found that the width of small blood vessels is three to four pixels, and that of thick blood vessels is seven to eight pixels. The detector is subdivided. After research, it is found that L <9 is divided into small scales, and the other is the best when the large scales.

In order to enhance the contrast between the small blood vessels and the background and improve the accuracy of small blood vessel segmentation, this article performs local enhancement on retinal blood vessel images.


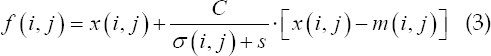


Where *x(i,j)* is the pixel value of a certain point, *m(i,j)* is the average value in the window, *f(i,j)* is the output pixel value, *σ(i,j)* is the standard deviation of the pixel value in the window, C is the control parameter, taking the experience value, s is a negligible value greater than zero, and 
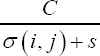
 is the local gain. Use equation (3) to locally enhance the inverted image.

In this paper, a 5 × 5 window is used as a mask. The greater the probability that the pixel values in the window are the same, the smaller *σ(i,j)* is, the larger 
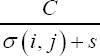
 is, and the local contrast is improved.

In order to highlight the main contours of the blood vessels in the retinal image, reduce noise, and suppress uneven illumination areas, this article enhances the retinal blood vessel images globally.


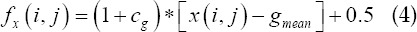


In the formula, *f(i,j)* is the pixel value of a point, *g_mean_* is the global average, *f_x_(i,j)* is the output pixel value, and *c_g_* is the control parameter, which is an empirical value. The inverse image is globally enhanced using equation.^4^

Global enhancement can suppress uneven illumination in small areas of the image, reduce background noise, and improve global contrast. Then, a large-scale linear detector is used to detect the globally enhanced image, and a large-scale response function is obtained. On a large scale, the number of pixels contained in the detection line L is large, which is conducive to the detection of thick blood vessels and global contours. The detection results have more prominent contours and less noise.

In order to retain the advantages of the detector at different scales, different response functions at small scale and large scale are fused.


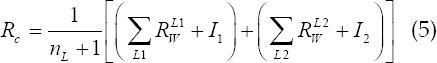


In the formula, L1 and L2 respectively correspond to different scales, and I1 and I2 are the images after local enhancement and global enhancement, respectively. Use (5) for linear fusion. This fusion method retains more blood vessel information, reduces the influence of background pixels, and is conducive to the extraction of small blood vessels. The final retinal blood vessel segmentation map is obtained after fusion.

## RESULTS

According to the evaluation criteria, pixels are divided into the following four categories: TP indicates the blood vessel pixels that are correctly segmented, also called true positives; FP indicates the blood vessel pixels that are incorrectly segmented, also called false positives; TN indicates the background points that are correctly segmented, called true negative; FN represents the background point that was incorrectly segmented, also called false negative. The ACC used in the evaluation criteria refers to the ratio of the sum of the number of vascular pixels and non-vascular pixels correctly judged to the sum of the number of vascular and non-vascular pixels in the standard image; TPR refers to the number of vascular pixels correctly judged The ratio of the number to the number of all vascular pixels in the standard image; FPR refers to the ratio of the number of vascular pixels that are incorrectly determined to the number of all non-vascular pixels in the standard image, as shown in Table-I.

**Table-I:** Classification performance.

*Segmentation result*	*Segmentation result*	*Incorrectly segmented pixels*
Vascular point	Vascular point	True positive (TP)
Background point	Background point	False negative (FN)
total	P	N

Its calculation formula is


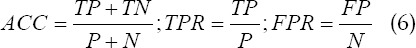


Using the above method to judge the segmentation effect, the larger the ACC and TPR, and the smaller the FPR, the better the segmentation effect and the higher the accuracy.

The algorithm and literature in this paper are verified on two public databases, STARE and DRIVE, and compared with standard image using formula (6). It can be seen that compared with standard images, the document segmentation results are better for the extraction of thick blood vessels, but they lack many small blood vessels, and the pixels of mis-segmentation. Many studies^7^ have compared with the literature, the number of pixels for mis-segmentation is reduced, but the accuracy of small blood vessel segmentation is low. Compared with the above two methods, the algorithm in this paper has segmented both vascular and non-vascular pixels very well. As a result, it contains more small blood vessels.

## DISCUSSION

As for the evaluation and analysis of the algorithm, an experiment was conducted on the images in the test set, with the manual segmentation results in set A as standard reference images. The STARE database contains 20 retinal images with a size of 605 pixels × 700 pixels. Also, manual segmentation is performed by two experts. In one expert’s segmentation result, blood vessels account for 10.4% of the total pixels, and it is 14.9% in the other expert’s result. The segmentation results of the first expert are used as standard reference images. It is noted that, there are lots of disparities in the segmentation results of the algorithm in reference;^8^ the segmentation results of the algorithm in reference^9^ omit a lot of small blood vessels, and these small blood vessels are of great significance to the analysis of retinal images. Compared with the above two methods, the segmentation results of the algorithm in this study display more small blood vessels, and the connectivity of the retinal vessel tree is better. Additionally, the retinal vessel connectivity in the segmentation result of the algorithm in reference 8 is poor; and the segmentation result of the algorithm in reference 9 contains many mis-segmented pixels, which reduces the segmentation accuracy. Compared with the above two methods, the segmentation results of the STARE database by the algorithm in this study show that, the retinal blood vessels are smooth, with good connectivity, and high accuracy. In order to analyze the connectivity of the segmentation results and the detection accuracy for branch intersections, the retinal blood vessels are skeletalized. The blood vessels extracted by the algorithm in this study have good connectivity, while in the segmentation results of the algorithms in reference 8 and reference 9, part of the blood vessel information is missing. In order to further compare the segmentation performance of these algorithms, the accuracy of the segmentation results is evaluated factoring into Accuracy (Acc), True positive rate (TPR), False positive rate, (FPR), and F-measure. Among them, the accuracy is the ratio of correctly segmented blood vessels and background pixels to pixels in the entire field of view; the true positive rate is the ratio of the total number of blood vessel pixels correctly segmented by the algorithm to the number of standard blood vessel pixels in the field of view, and this ratio can also represent the recall rate (Recall); the false positive rate is the ratio of the number of blood vessel pixels incorrectly segmented by the algorithm to the number of standard background pixels in the field of view; and the F-measure characterizes the relationship between the precision of the algorithm and the recall rate, where the precision is the ratio of the number of blood vessels correctly segmented by the algorithm to the total number of segmented blood vessels.^10^-^12^

The research of retinal blood vessel image technology is in the ascendant. Although this study has achieved good results in segmenting retinal blood vessels, a lot of improvement is needed^13^-^15^. The experimental samples are all from foreign databases of retinal images, which affects the universality of the study 16,17. Therefore, more fundus retinal images are needed and whether satisfactory results can be achieved using the algorithm in the study will be further explored. Further, in the diseased retina image, because there is much noise, the contrast between the blood vessel and the background is low. Therefore, it is necessary to use the algorithm in this study to process the diseased retina image. This study only deals with retinal blood vessels, and other tissue of fundus images, such as the macula and optic disc, are also important for diagnosing diseases. How to realize automatic segmentation and extraction of these tissue will be studied in the future.

## CONCLUSIONS

This paper proposes an effective retinal blood vessel segmentation method. This method performs linear and small-scale and large-scale linear detection on the locally and globally enhanced images, respectively. The local enhancement magnifies the differences in local regions and uses small-scale Vessel thin sections are detected to preserve detailed information; global enhancement improves global contrast, and the large-scale detection line contains many pixels, which makes the overall outline of the blood vessel prominent and less noisy. Finally, image fusion is performed to preserve the advantages of different scales. The experimental results show that the algorithm in this paper improves the sensitivity to small blood vessels, and its accuracy and true positive rate are significantly improved. At the same time, it has high robustness to noise and has high practical value.

### Author`s Contribution:

**YH** conceived the study, literature review, data analysis, helped to draft the manuscript .

**HX** helped in design, data collection, article drafting & critical revision.

**RQ** takes the responsibility and is accountable for all aspects of the work in ensuring that questions related to the accuracy or integrity of any part of the work are appropriately investigated and resolved.
